# Complete mitochondrial genome of the Korean endemic species *Coreoleuciscus aeruginos* holotype from Korea (Cypriniformes, Cyprinidae)

**DOI:** 10.1080/23802359.2021.1902415

**Published:** 2021-03-24

**Authors:** Kang-Rae Kim, Ha-Yoon Song, Yong Hwi Kim, Jong Yeon Park, In-Chul Bang

**Affiliations:** a Department of Life Science & Biotechnology, Soonchunhyang University, Asan, Republic of Korea; b Inland Fisheries Research Institute, National Institute of Fisheries Science, Gapyeong, Republic of Korea

**Keywords:** Complete mitochondrial genome, Cyprinidae, *Coreoleuciscus aeruginos*, endemic fish

## Abstract

This study is the first to research report the complete 16,563 bp mitochondrial genome of *Coreoleuciscus aeruginos*, which consists of 13 protein-coding genes (PCGs), 2 ribosomal RNA (rRNA) genes, 22 transfer RNA (tRNA) genes, and a control region (D-loop). The overall base composition of the complete genome is 31.0% A, 28.04% T, 16.27% G, and 24.69% C, with a high A + T content of 59.04%. According to our phylogenetic analysis, *C. aeruginos* is most closely related to *Coreoleuciscus splendidus*.

*Coreoleuciscus aeruginos* (family Cyrinidae) is an endemic Korean freshwater fish distributed in riffles of the middle and upper reaches of the Seomjingang and Nakdonggang River systems. A single *C. aeruginos* specimen was previously reported as *C. splendidus* (Mori 1935), but was later described as a new species with significant morphological and genetic differences from *Coreoleuciscus splendidus* (Song and Bang 2015). This study first reports the complete mitogenome of the *C. aeruginos* holotype and provides the phylogenetic location of genus *Coreoleuciscus*.

In December 2009, a single *C. aeruginos* holotype specimen was collected from Deokcheon River in the Nakdonggang River system in Korea (35°16′29N, 127°50′33E). The holotype specimen (voucher number SUC-1290) is stored in the specimen room, Department of Life Science & Biotechnology, College of Natural Sciences, Soonchunhyang University, Asan City, Chungcheongnam-do, Republic of Korea. Genomic DNA was extracted from the caudal fin of the specimen using a Genomic DNA Prep Kit (Biofact, Daejeon, Korea). Genomic DNA was stored in a freezer at −80 °C in the specimen room of Soonchunhyang University. The complete mitogenome sequence was prepared using the MGI Easy DNA Library Prep Kit (MGI, Shenzhen, China), and a DNA library consisting of 150 bp pair-end reads was obtained and then sequenced by using the MGISEQ-2000 platform (MGI). The raw data were cleaned using the Cutadapt version 1.9 program (Martin 2011) and assembled using the Geneious version 11.0.3 program. The assembled sequence was annotated using the MITOS Web Server (Bernt et al. 2013). Finally, the complete mitochondrial DNA sequence of *C. aeruginos* was deposited into GenBank (accession no. MW192440).

The complete mitochondrial genome of *C. aeruginos* is 16,563 bp, containing 13 protein-coding genes (PCGs), 2 ribosomal RNA genes (rRNA), 22 transfer RNA (tRNA) genes, and a control region (D-loop). Only the *CO1* gene is started with GTG; the remaining 12 PCGs are started with ATG. Three PCGs (*CO2*, *Cytb*, and *ND2*) are terminated with an incomplete T stop codon, and the two PCGs (*CO3* and *ND4*) are terminated with TA. Eight of the PCGs (*CO1*, *ATP6*, *ATP8*, *ND1*, *ND3*, *ND4L*, *ND5*, and *ND6*) are ended with a complete TAA or TAG. For two of the tRNA genes (*Gly* and *Arg*), the A + G and C + T ratios are the same. Two tRNA genes (*Ser2* and *Glu*), two rRNA genes (16S rRNA and 12S rRNA), and the *ND6* gene are located on heavy strands. Twelve PCGs (*ND1*, *ND2*, *ND3*, *ND4*, *ND4L*, *ND5*, *ATP6*, *ATP8*, *CO1*, *CO2*, *CO3*, and *Cytb*) and 10 tRNA genes (*Gln*, *Met*, *Ala*, *Asn*, *Cys*, *Tyr*, *Ser*, *Asp*, *Thr*, and *Pro*) are located on the light strands.

The overall base composition of the *C. aeruginos* genome is 31.0% A, 28.04% T, 16.27% G, and 24.69% C, with a high A + T content of 59.04%. The rRNA of *C. aeruginos* consists of 16S rRNA (1693 bp) and 12S rRNA (958 bp); 16S rRNA is located between tRNA *Val* and tRNA *Leu*, and 12S rRNA is located between tRNA *Phe* and tRNA *Val*.

The phylogenetic tree was constructed and analyzed via the maximum likelihood and Bayesian inference methods using the PhyML version 3.1 (Guindon et al. 2010) and MrBayes version 3.2.7 (Ronquist et al. 2012) programs, respectively. We applied a TIM3+ I + R model to the data based on 13 PCG sequences of a total of 18 species (Guindon and Gascuel 2003; Darriba et al. 2012). The results showed that *C. aeruginos* was most closely related to *C. splendidus* (Figure 1). In the phylogenetic tree, *C. aeruginos* was clustered with two *C. splendidus* sequences (JN831358 and DQ347951). Two sequences collected from the Seomjingang and Nakdonggang River had been registered in the National Center for Biotechnology Information (NCBI) database as *C. splendidus* (JN831358 and DQ347951); however, the former was later identified as a new species (*C. aeruginos*). Therefore, we presume that both DQ347951 and JN831358 are actually sequences of *C. aeruginos*. Thus, *C. aeruginos* formed a single clade in the phylogenetic tree, which clearly separated into *C. aeruginos* and *C. splendidus*.

## Data Availability

The genome sequence data that support the findings of this study are openly available in GenBank of NCBI at (https://www.ncbi.nlm.nih.gov/) under the accession no. MW192440. The associated BioProject, SRA, and Bio-Sample numbers are PRJNA688383, SRX9785594 and SAMN17174017, respectively. The phylogenetic tree constructed maximum likelihood and Bayesian inference based on 13 PCGs. The numbers above the nodes represent the bootstrap support value (left) and probability value (right) for each branch. The scientific names are followed by their GenBank accession numbers.
